# Correction to: Student Summer Research Awards – SSRA Abstracts of the SSRA September 2023 meeting, UCD School of Medicine

**DOI:** 10.1007/s11845-024-03746-x

**Published:** 2024-07-06

**Authors:** Edward Moore

**Affiliations:** Health Sciences, University College Dublin, Belfield, Dublin 4 Ireland

**Correction to: Irish Journal of Medical Science (2024) 193 (Suppl 2):S61–S101**

10.1007/s11845-024-03683-9

These abstracts were initially published as non-open access despite being intended for open access publication. This issue has now been resolved.

The original article has been corrected.

